# Automation of an integrated micro‐scale platform for monoclonal antibody process development by incorporation of a depth filter mimic

**DOI:** 10.1002/btpr.70077

**Published:** 2025-10-03

**Authors:** Paras Sharma, Petra Sebastian, Lars Robbel, Michael Schmitt, Daniel G. Bracewell

**Affiliations:** ^1^ Department of Biochemical Engineering University College London London UK; ^2^ CSL Behring Innovation Marburg Germany

**Keywords:** downstream processing, high throughput process development, micro‐scale, monoclonal antibodies, scale‐down

## Abstract

High throughput process development (HTPD) has been widely adopted for efficient development and optimization of chromatographic operations in monoclonal antibody (mAb) purification. However, the integration of non‐chromatographic unit operations, particularly depth filtration following protein A chromatography, which is essential for the removal of process‐ and product‐related impurities prior to the ion exchange chromatography (IEX) operations, remains a challenge due to the absence of commercially available micro‐scale depth filtration tools. This limits the integration of this unit operation within the purification sequence, restricting the analysis of process interactions and overall process understanding. In this study, a micro‐scale HTPD platform was designed and evaluated to enable integration of a depth filtration mimic, Sartobind® Q anion exchange adsorber, within a mAb purification sequence. This was achieved by translating laboratory‐scale protocols to the micro‐scale using workflow design tools and executed on an automated liquid handling system. Step yields and impurity clearance were assessed to confirm the equivalence of scale‐down. The Sartobind® Q membrane achieved effective removal of host cell DNA (hcDNA), while subsequent IEX operations removed host cell proteins (HCPs) and high molecular weight components (HMWC), meeting target product quality specifications. The platform demonstrated robustness across varying impurity profiles, supporting its applicability for diverse process intermediates. Comparative analysis with laboratory‐scale operations confirmed the performance and scalability of the micro‐scale system, reducing the total run time by greater than 50%. The integrated HTPD platform offers a resource‐efficient, scalable approach for comprehensive mAb purification process development and is suitable for developability assessments during early‐stage development.

## Graphical abstract

Integrating non‐chromatographic operations, such as low pH viral inactivation and a depth filtration mimic, into an automated micro‐scale platform enhances productivity while reducing resource and time requirements. By facilitating rapid, data‐rich experimentation, the platform offers a powerful and efficient tool for assessing and optimizing monoclonal antibody (mAb) production processes.

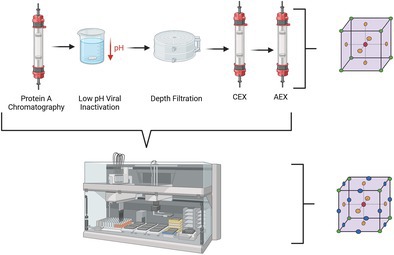

## 1 INTRODUCTION

The success of monoclonal antibodies (mAbs) for clinical and diagnostic applications has created a need to develop economic, efficient, and scalable production processes. Therefore, an increase in efficiency and a decrease in time and costs is a priority. Within the past three decades, significant advancements have been made in cell culture processing, which has allowed productivity to increase 100‐fold.^1^, ^2^ The rise in productivity brings economies of scale to upstream processing,^3^ which puts pressure on existing downstream purification processes to isolate the target protein from the complex harvest material that constitutes product‐related impurities, process‐related impurities, and contaminants. Higher titre not only correlates with increased impurities, which will exhaust existing capacities, but will also shift the costs of manufacturing towards downstream processing, as economies of scale do not translate.^4^ This highlights both the capacity and impurity removal challenges to downstream processing, accentuating the need for new approaches to optimization strategies and technology development.^1^

Consequently, high throughput process development (HTPD) has been employed to accelerate development and optimization, particularly for mAb downstream processes. Time‐ and resource‐efficient process development can be achieved by leveraging the miniaturization, automation, and parallelization capabilities of HTPD strategies. This has been well documented for the demonstration, development, and optimization of a range of chromatographic operations.^5^, ^6^, ^7^, ^8^, ^9^, ^10^ However, employment beyond chromatographic operations is not widely established. This prevents the integration of non‐chromatographic operations within the purification sequence, and therefore, the investigation of process intermediates. Consequently, our previous research focused on the integration of the protein A chromatography and low pH viral inactivation (VI) operations onto an automated micro‐scale platform.^11^ The investigative power of the platform was demonstrated and presents the productivity gains when leveraging HTPD beyond chromatographic operations. This suggests that even greater gains can be achieved through the integration of further unit operations within the mAb purification sequence.

The purification of mAbs is typically accomplished by protein A affinity chromatography, followed by viral inactivation and two additional ion exchange chromatography (IEX) operations.^12^ Depth filtration, which is traditionally used for clarification of cell culture broths during primary recovery, is also employed immediately after protein A chromatography and low pH VI, preceding the polishing stage.^13^ This is required for removal of process contaminants and impurities, such as host cell proteins (HCPs), host cell DNA (hcDNA), and aggregates, which are important to protect and maintain the capacity of the proceeding IEX operations.^14^, ^15^, ^16^ Fouling of chromatographic resin poses a challenge to such purification processes, which can cause a considerable reduction in binding capacities.^17^ This may impact the separation ability of the operation and result in a product that is not of the required output quality, while increasing manufacturing costs due to a reduction in column lifetime. Consequently, it is imperative that the removal of process contaminants and impurities is sufficient prior to IEX.

A typical depth filter takes the form of a disc or sheet that comprises three components.^18^ Cellulose or polypropylene fibers, with a characteristic pore size, serve as the base of the depth filter and enable filtration of particulates based on size. The next component, the filter aid, contributes to the capture of particulates, increasing the throughput of the depth filter. Filter aids are commonly siliceous materials, for example, diatomaceous earth (DE), fossilized diatoms, and perlite.^18^ Finally, a polymeric binding agent carries a level of cationic charge.^19^ The several components comprising a depth filter provide the functionality for particulate removal through size exclusion and adsorption of soluble impurities through electrostatic attraction and hydrophobic interactions.^19^ Consequently, depth filtration has been extensively used for the removal of a multitude of process contaminants and impurities and has also been established for post‐protein A affinity chromatography and low pH VI filtration for IEX material preparation. For example, positively charged depth filters were utilized for the removal of *Escherichia coli*‐derived and several other endogenous endotoxins.^20^ Zeta Plus® VR series depth filters have previously been employed for the removal of enveloped retrovirus and non‐enveloped parvovirus by adsorption.^21^ Charged depth filters have also been used for hcDNA clearance, where it was demonstrated that the level of charge is correlated with the extent of DNA removal.^13^ It has also been shown that not only electrostatic interactions but hydrophobic interactions are fundamental to the reduction of hcDNA.^22^, ^23^

Despite being a ubiquitous operation for mAb processing, the availability of micro‐scale depth filters is still lacking to the best of our knowledge. This is primarily due to the complexity of the depth filter media.^18^ The material requirements, along with the operational constraints, have hindered the development of micro‐scale models. This also prevents the integration of the depth filtration operation within the micro‐scale purification sequence, and therefore, the investigation of process intermediates. Consequently, an alternative method that can achieve sufficient impurity clearance, comparable to that of a laboratory‐scale depth filter, while being integrated onto an automated micro‐scale platform, is to be sought. Current research has focused on the development of high throughput depth filtration using ultra scale‐down (USD) technology, with the aim of increasing parallelization and decreasing resource and labor requirements.^18^ Progress has been made in the establishment of small‐scale filtration. For example, Rayat et al. demonstrated the scale down of crossflow microfiltration through the design and implementation of an ultra‐scale down (USD) system that was not only compatible with an automatic liquid handler but also emulated the geometric and hydrodynamic properties of large‐scale operations.^24^ Furthermore, Lau et al. utilized an existing USD device by Jackson et al. for the removal of impurities by depth filtration, as well as for evaluating changes in filter resistance.^25^, ^26^ While this presents an approach to use HT technologies to evaluate depth filtration at the micro‐scale for development and optimization, these USD devices are not commercially available and have only been developed for research purposes.

Consequently, this study investigates the use of an anion exchange membrane adsorber to purify the load material in preparation for the subsequent IEX operation. The Sartobind® Q has been selected as the micro‐scale technology due to its expected comparable functionality for particulate removal as a laboratory‐scale depth filters.^17^ Due to the regenerated and stabilized cellulose base material, as well as the ion exchange ligands attached to the membrane, separation by Sartobind® Q is achieved based on charges carried by solvent molecules, akin to atypical laboratory‐scale size‐ and charge‐based depth filters composed of cellulose fibers and DE.^27^ Following scale comparison between the material output quality of the micro‐scale and laboratory‐scale intermediates, the Sartobind® Q operation is then integrated with the preceding low pH VI and proceeding IEX operations to establish a fully automated micro‐scale platform for monoclonal antibody purification.

**FIGURE 1:**
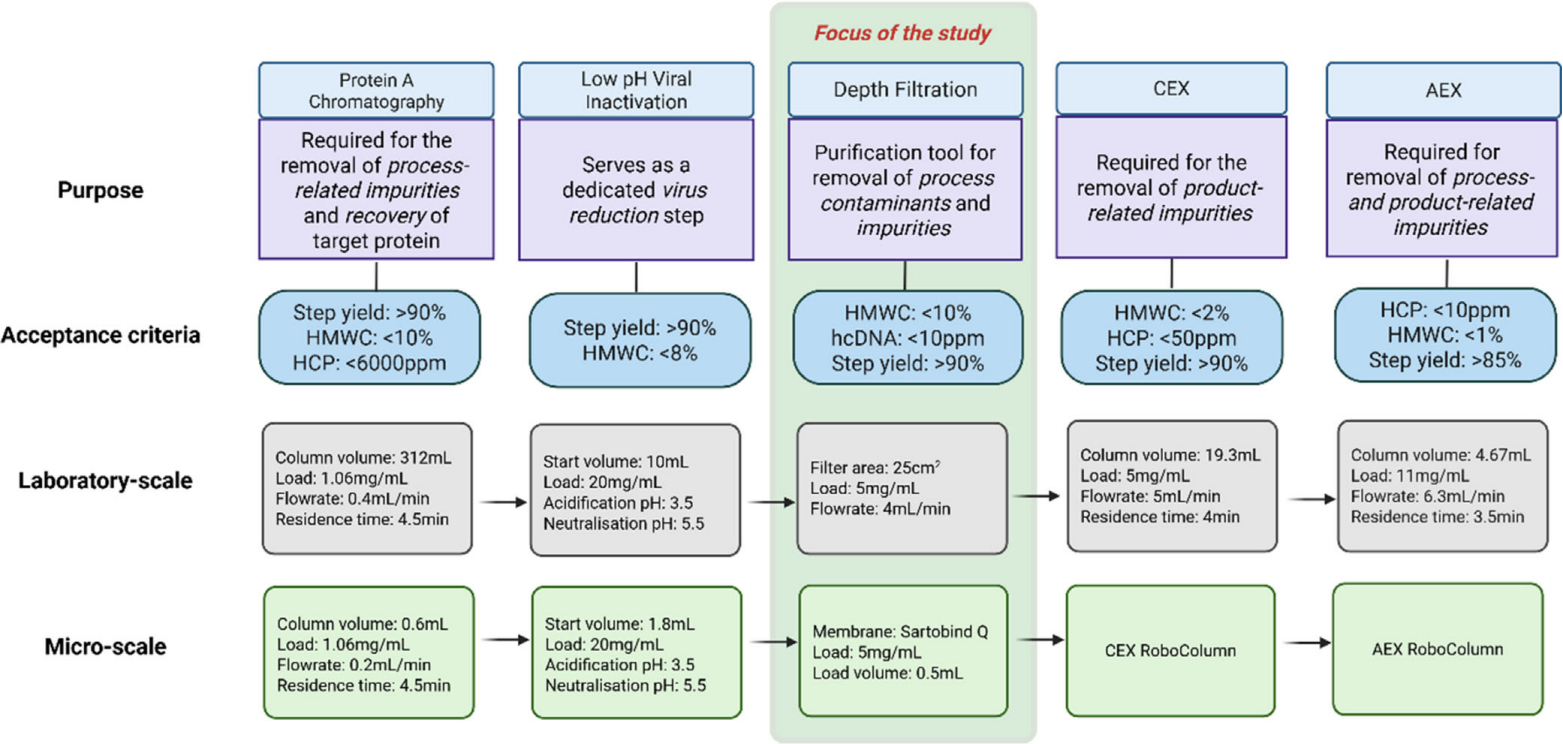
Process flowsheet of a monoclonal antibody downstream process into which Sartobind® Q operation will be integrated within. The purification sequence highlighted in gray and green represent the laboratory‐scale and micro‐scale, respectively, with the specified process parameters for each unit operation. The objective of each operation is also presented which emphasizes the importance of each within the sequence. The critical quality attributes (CQAs) for each micro‐scale operation are shown. These form the criteria for which the output material must satisfy to demonstrate the equivalence between the micro‐scale and laboratory‐scale unit operations. The operation highlighted in green represent those which are investigated in this study. Figure created with BioRender.com .

The scale transfer was achieved by translating laboratory‐scale workflows into automated workflows using the Python package, Robotools (v1.11.2), before executing on the Tecan Freedom Evo 200. This platform was then used as a tool to assess the output quality of process intermediates with different impurity profiles. This paper also discusses the economic advantages of integrating this operation within the mAb purification sequence (Figure 1) to increase process understanding and inform on process development.

## 2 MATERIALS AND METHODS

### 2.1 Material source

The material source is clarified harvest of a fully human IgG1κ monoclonal antibody produced in cultures of CHO cells.

### 2.2 Micro‐scale operations

#### 2.2.1 Protein A chromatography

This operation utilized 8 OPUS® RoboColumns® (Repligen, United States) pre‐packed with MabSelect SuRe resin (Cytiva, United States). The column parameters are presented by Table 1.

**TABLE 1:** OPUS® RoboColumn® MabSelect SuRe column parameters used for the micro‐scale protein A operation.

Column	Inner diameter	Gel bed height	CV	Load concentration	Load capacity	Residence time
	[cm]	[cm]	[mL]	[mg/mL]	[mg/mL]	[min]
MabSelect SuRe	0.50	3.00	0.59	1.06	25.00	4.05

The starting material for this operation was 13.9 mL of clarified harvest per column (1.06 g/L load), which was thawed in a water bath at 25°C, before being placed, along with the buffers, onto the deck of the Tecan Freedom EVO® 200 (Tecan, Switzerland).

Once the consumables were defined in the Robotools (v1.11.2) script and matched to the deck layout created in Freedom EVOware (Tecan, Switzerland), the workflow was designed and created, which was based upon the existing laboratory‐scale operation.^28^ The workflow was then exported as a series of commands to Freedom EVOware (Tecan, Switzerland). The implementation of the micro‐scale chromatographic operations closely mimics that of the laboratory‐scale operation across the protocol steps, including (1) equilibration; (2) load; (3) post‐load wash; (4) wash; (5) elution; (6) regeneration; (7) sanitisation; (8) re‐equilibration; and (9) storage. However, the experimental setup differs between the two since the transfer of solutions to the RoboColumns® (Repligen, United States) is conducted in a discrete, as opposed to continuous manner. Therefore, material is aspirated within and between the columns by each of the eight channels of the Liquid Handling Arm (LiHa), which plays the role of a simple inlet and outlet pump. The Robot Manipulator Arm (RoMa) is responsible for moving specific plates to defined locations on the deck, for example, movement of the 96 deep‐well plate for collection of the eluted fractions. In the micro‐scale format, where in‐line UV detection was not available, pooling was instead standardized to a fixed fraction of the eluate, typically corresponding to 4 CV, consistent with the laboratory‐scale operation.

#### 2.2.2 Low pH viral inactivation

The low pH VI operation was designed and constructed using Freedom EVOware (v2.8 SP7) (Tecan, Switzerland), the protocol of which was based upon the existing laboratory‐scale operation.^28^ The workflow was then executed, in sequence with the preceding protein A chromatography operation, on the Tecan Freedom EVO® 200 (Tecan, Switzerland).

The actions performed by the robotic station involved diluting the pooled protein A eluate 1:4 with the protein A elution buffer (50 mM acetate, pH 3.8) to a target conductivity of ≤0.5 mS/cm, prior to the low pH hold. The volume of acidification solution (3 M acetic acid) added to the diluted protein A eluate was adjusted depending on the desired pH to be achieved for low pH hold. For example, ~10%–12% (v/v) of start material amount was added to achieve a pH of 3.5 through acidification solution, and ~14%–15% (v/v) of start material amount was added to achieve a pH of 5.5 through neutralization solution (2 M tris). Following acidification, the eluate was held for 45 min before the addition of neutralization solution. The pH of the samples was measured on‐deck using the LV1R‐1951‐01_2 sensor spots (PreSens, Germany) following acidification and neutralization.

Mixing efficiency at the micro‐scale was assessed using a colourimetric dye assay with bromophenol blue, designed to evaluate the PostMix liquid handling policy when executed on the Tecan Freedom EVO® 200 platform (Tecan, Switzerland). The PostMix protocol consisted of three aspirate‐dispense cycles applied to the target solution. Bromophenol blue was selected as a pH‐sensitive indicator due to its distinct color transition from yellow at pH <3 to purple at pH >4.6. Following buffer addition to adjust the solution pH, mixing was performed according to the PostMix policy. Absorbance at 590 nm was recorded using a microplate reader 60 s after mixing initiation. To ensure representative sampling, aliquots were collected from three discrete locations within the well and averaged.

Mixing homogeneity was quantified using the dimensionless parameter M60:

(1) $$M_{60}=\frac{A590_{60s}}{A590_{\infty }}$$

where A590,60s represents the absorbance measured 60 s after mixing, and A590, ∞ corresponds to the absorbance of a fully mixed reference. This assay served as a basis for assessing the suitability of existing mixing strategies for the low pH VI step. Based on the comparison, the liquid policy was identified as an appropriate method for preparing the required solutions.

#### 2.2.3 Sartobind Q workflow design and task execution

**FIGURE 2:**
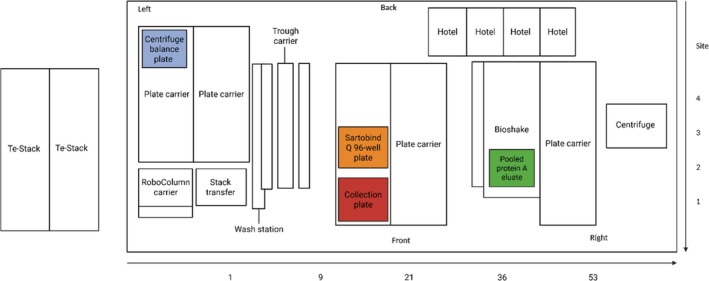
The Tecan Freedom EVO® 200 deck configuration for the implementation of the micro‐scale Sartobind Q operation. The *x*‐axis presents the locations of the labware and carriers on the deck, including the labware required for the Sartobind® Q operation, centrifuge balance plate (highlighted in blue), Sartobind® Q 96‐well plate (highlighted in orange), 96 deep‐well collection plate (highlighted in red), 200 mL trough containing the pooled protein A eluate (highlighted in green). The *y*‐axis corresponds to the site number, which is required for navigation to the specific labware by the Liquid Handling Arm (LiHa) and two Robot Manipulator Arms (RoMa). The typical path of the LiHa during the Sarotbind® Q operation involves movement of liquid between the wash station, trough carrier, plater carrier and Bioshake. The typical path of the RoMa during the Sartobind® Q operation involves movement of the plates between the plate carrier, Te‐Stack and centrifuge. Figure created with BioRender.com .

The micro‐scale Sartobind Q operation was designed and constructed using Robotools, the protocol of which was developed specifically for the integrated micro‐scale platform. Once successfully designed and tested, the workflows were then executed on the Tecan Freedom EVO 200 (Tecan, Switzerland), in sequence with the preceding low pH VI operation. The deck layout used for the operation is presented by Figure 2.

The actions of the robotic liquid handler involve (1) equilibration; (2) load; (3) adjustment of the centrifuge balance plate; (4) centrifugation; (5) shaking and collection of flowthrough; (5) wash; (7) adjustment of the centrifuge balance plate; (8) centrifugation; (9) shaking and collection of wash, and (10) regeneration. Following the operation, the collection plates are stored in the Te‐Stack and can be removed for material analysis, while the downstream operation commences.

#### 2.2.4 Cation exchange chromatography

This operation utilized 8 OPUS® RoboColumns® (Repligen, United States) pre‐packed with a commercially available CEX resin (Cytiva, United States). The starting material for this operation was 20 mL of the Sartobind® Q flowthrough per column, which was placed, along with the pre‐prepared buffers, onto the deck of the Tecan Freedom EVO® 200 (Tecan, Switzerland).

Once the consumables were defined in the Robotools (v1.11.2) script and matched to the deck layout created in Freedom EVOware (Tecan, Switzerland), the workflow was designed and created, which was based upon the existing laboratory‐scale operation.^28^ The workflow was then exported as a series of commands to Freedom EVOware (Tecan, Switzerland). The actions performed by the robotic liquid handler include (1) equilibration; (2) load; (3) wash; (4) gradient elution; (5) regeneration; (6) re‐equilibration; (7) neutralization, and (8) storage. Elution was performed using a gradient of increasing conductivity. As automated liquid handlers operate in a discrete rather than continuous manner, a pseudo‐linear gradient was implemented by increasing the NaCl concentration from 15 mM to 150 mM across 12 incremental steps. The gradient profile was pre‐calculated based on the total number of steps, and the corresponding buffers were prepared in advance and positioned on the deck prior to operation.

#### 2.2.5 Anion exchange chromatography

This operation utilized 8 OPUS® RoboColumns® (Repligen, United States) pre‐packed with a commercially available AEX resin (Cytiva, United States). The starting material for this operation was 7 mL of the Sartobind® Q flowthrough CEX eluate per column, which was placed, along with the pre‐prepared buffers, onto the deck of the Tecan Freedom EVO® 200 (Tecan, Switzerland).

Once the consumables were defined in the Robotools (v1.11.2) script and matched to the deck layout created in Freedom EVOware (Tecan, Switzerland), the workflow was designed and created, which was based upon the existing laboratory‐scale operation.^28^ The workflow was then exported as a series of commands to Freedom EVOware (Tecan, Switzerland). The actions performed by the robotic liquid handler include (1) equilibration; (2) load; (3) post load wash; (4) stripping; (5) regeneration; (6) sanitization; (7) neutralization; and (8) storage. In the micro‐scale format, where in‐line UV detection was not available, pooling was standardized to a fixed fraction of the eluate. A collection volume of approximately 4 CV was selected, reflecting the typical elution profile observed at laboratory scale and ensuring consistency between the two formats.

### 2.3 Laboratory‐scale operations

#### 2.3.1 Protein A chromatography

The laboratory‐scale protein A chromatography operation was conducted using the ÄKTA Avant 150 chromatography system (Cytiva, United States). An OPUS® ValiChrom® was pre‐packed with MabSelect SuRe (Cytiva, United States). The column parameters are presented in Table 2.

**TABLE 2:** MabSelect SuRe column parameters used for the laboratory‐scale protein A chromatography operation.

Column	Inner diameter	Gel bed height	CV	Load concentration	Load capacity	Residence time
	[cm]	[cm]	[mL]	[mg/mL]	[mg/mL]	[min]
MabSelect SuRe	4.40	20.50	312	1.06	25.00	4.50

The Protein A chromatography method was configured in UNICORN™ v7.6 (Cytiva, United States) based on a laboratory‐scale process flowsheet (Figure 1) and manufacturer recommendations for resin operation, ensuring consistency with the micro‐scale set‐up (with the exception of gel bed height). For laboratory‐scale operation, peak collection was triggered once the absorbance at 280 nm (A280) rose above baseline and continued until it decreased to 20 mAU/mm path length. This elution profile corresponded to 4 CV, which was therefore defined as the collection criterion for the micro‐scale operation.

#### 2.3.2 Low pH viral inactivation

The laboratory‐scale low pH VI step firstly involved removing 10 mL of the protein A eluate (~20 mg/mL protein concentration) from storage and thawing in a water bath at 25. Following this, the eluate was diluted with 50 mM acetate, pH 3.8, to a target conductivity of ≤0.5 mS/cm. The pH was decreased using acidification solution (3 M acetic acid), with stirring at 115 revolutions per minute (rpm), over a period of ≥15 min to pH 3.5 ± 0.1. The eluate was then held for 45 min, before addition of neutralization solution (2 M Tris) to a pH of 5.5 ± 0.1 with stirring of 115 rpm, over a period of ≥15 min.

#### 2.3.3 Depth filtration

Following low pH VI, the protein A eluate was then filtered using the laboratory‐scale Depth Filter with an effective filter area of 25cm^2^ and a load of 28.5 mg/m^2^, operated at a flowrate between 4‐7 mL/min. This was performed using the ÄKTA Avant 150 chromatography system (Cytiva, United States), with the method constructed on UNICORN™ (v7.6) (Cytiva, United States).

The virus inactivated protein A eluate was then filtered using a Sartopore® 2 0.45/0.22 μm capsule filter (Sartorious, Germany). The flowrate of this operation was consistent with that of depth filtration. Pre‐flush of the Sartopore® 2 0.45/0.22 μm was not performed.

#### 2.3.4 Cation exchange chromatography

The laboratory‐scale CEX operation was conducted using the ÄKTA Avant 150 chromatography system (Cytiva, United States). An OPUS® ValiChrom® (Repligen, United States) column was pre‐packed with a commercially available CEX resin (Cytiva, United States). The column parameters are presented in Table 3.

**TABLE 3:** CEX column parameters used for the laboratory‐scale cation exchange chromatography operation.

Column	Inner diameter	Gel bed height	CV	Load concentration	Load capacity	Residence time
	[cm]	[cm]	[mL]	[mg/mL]	[mg/mL]	[min]
CEX	1.13	20	19.29	5.00	25.00	4.00

The CEX method was configured in UNICORN™ v7.6 (Cytiva, United States) based on a laboratory‐scale process flowsheet (Figure 1) and manufacturer recommendations for resin operation, ensuring consistency with the micro‐scale set‐up. Gradient elution was performed by increasing the NaCl concentration from 15 to 150 mM over 12 CV. Peak collection was initiated once the absorbance at 280 nm (A280) rose above baseline and continued until it decreased to 100 mAU/mm path length. This elution profile typically corresponded to a defined number of column volumes, which was therefore adopted as the collection criterion for the micro‐scale operation.

#### 2.3.5 Anion exchange chromatography

The laboratory‐scale AEX operation was conducted using the ÄKTA Avant 150 chromatography system (Cytiva, United States). A HiScreen™ column (Cytiva, United States) was pre‐packed with a commercially available AEX resin (Cytiva, United States). The column parameters are presented in Table 4.

**TABLE 4:** AEX column parameters used for the laboratory‐scale anion exchange chromatography operation.

Column	Inner diameter	Gel bed height	CV	Load concentration	Load capacity	Residence time
	[cm]	[cm]	[mL]	[mg/mL]	[mg/mL]	[min]
AEX	0.77	10	4.67	11.00	25.00	3.50

The AEX method was configured in UNICORN™ v7.6 (Cytiva, United States) based on a laboratory‐scale process flowsheet (Figure 1) and manufacturer recommendations for resin operation, ensuring consistency with the micro‐scale set‐up. The method was operated in flowthrough mode, with peak collection initiated once the absorbance at 280 nm (A280) rose above baseline and continuing until it decreased to 50 mAU/mm path length. This elution profile typically corresponded to 4 CV, which was therefore adopted as the collection criterion for the micro‐scale operation.

### 2.4 Dynamic binding capacity

Dynamic binding capacity was determined through breakthrough curve analysis to evaluate the retention of hcDNA on the Sartobind® Q membrane adsorber. A total of 50 mL of low pH VI process intermediate containing hcDNA was loaded onto the membrane. The Sartobind® Q membrane used in this study comprises multiple wells, each with a maximum volume capacity of 0.5 mL. Therefore, sample loading required sequential centrifugation steps. Each aliquot was applied to the membrane and centrifuged at 180×*g* for 2 min to facilitate passage through the membrane and collect the effluent. Effluent samples were collected and analyzed for absorbance at 280 nm using a Lunatic UV–Vis Spectrophotometer (Unchained Labs, United States) to generate the breakthrough curve. The breakthrough point was defined as the effluent concentration (*C*) reaching 10% of the inlet concentration (*C*_0_), and was calculated using the following equation:

(2) $${\mathrm{DBC}}_{10}=\frac{V_{10}*C_{0}}{V_{c}}$$

where *V*_10_ is the volume of feed loaded at 10% breakthrough (mL), *C*_0_ is the concentration of hcDNA in the feed (mg/mL), and V_c_ is the volume of the membrane (mL).

### 2.5 Analytical testing

#### 2.5.1 Host cell protein quantification

HCP quantification was performed using the HTRF CHO HCP Detection Kit (Revvity, United States), following the manufacturer's protocol with prefilled assay plates. An extended standard curve (0–1000 ng/mL) was generated using high‐concentration CHO HCP antigen (Cygnus Technologies, United States). Initial dilutions (1:10–1:10,000) were selected based on estimated HCP content, followed by two 1:2 serial dilutions. Standards and controls were also serially diluted. Standards included: 1000, 703, 301, 100, 33, 11, 4.0, and 0 ng/mL. Controls were diluted 1:100 and 1:200. Plates were incubated for 15 h at room temperature and read using a Spark reader (Tecan, Switzerland). Samples were tested in duplicate across three dilutions (six points/sample); controls across two dilutions (four points/control). Data analysis was performed using Magellan (Tecan, Switzerland), based on 665/620 nm fluorescence ratios.

#### 2.5.2 Host cell DNA quantification

HcDNA concentrations were determined using the Qubit™ dsDNA High Sensitivity (HS) Assay Kit (Thermo Fisher Scientific, United States). The reagent solution was prepared by diluting the Qubit™ HS reagent 1:200 in the HS buffer. A standard curve was generated using the two DNA standards supplied with the kit. For preparation of the samples, each were diluted with the HS buffer according to the expected concentration, before adding 1–10 μL of clarified protein solution to Qubit assay tubes containing 190–199 μL of working solution, ensuring that the total volume was 200 μL. Samples and standards were vortexed briefly and incubated at room temperature for 2 minutes prior to measurement. Fluorescence was measured using a Hamilton Microlab® STAR (Hamilton Company, United Kingdom) and hcDNA concentrations were calculated based on the standard curve using Magellan™ (Tecan, Switzerland). All measurements were performed in triplicate, and sample dilutions were applied as necessary to ensure values fell within the assay's dynamic range.

#### 2.5.3 Molecular weight quantification

The presence of molecular weight content was determined by size exclusion‐high performance liquid chromatography (SE‐HPLC). This was performed using a Vanquish HPLC System (Agilent Technologies, United States) with an silica‐based SEC column in an isocratic elution method. The mobile phase consisted of either 100 mM Bis‐Tris propane, 220 mM NaCl, pH 7.0 or 500 mM Bis‐Tris propane, 1100 mM NaCl, pH 7.0, both of which were diluted 5‐fold prior to use. The flow rate was set to 0.3 mL/min, and the total run time was 12 min. The column was maintained at 35°C, and the autosampler was held at 6°C. Samples were injected using a 25 μL sample loop, with an injection volume range of 0.2–25 μL based on the protein concentration. A target column load of 20 μg total protein was used per injection. UV detection was performed at 280 nm with a 5 Hz data collection rate, 1‐s response time, and a peak width setting of 0.1 min using a 2.5 μL flow cell.

### 2.6 Statistical analysis

#### 2.6.1 Student's *t*‐test

Student's *t*‐test was applied to compare the means of two independent groups under the assumption of equal variances and approximately normal distributions. The null hypothesis (*H*_0_) stated that there was no significant difference between the paired groups, while the alternative hypothesis (*H*₁) indicated that a statistically significant difference existed. The test statistic was calculated as:

(3) $$t=\frac{{\bar{x}}_{1}-{\bar{x}}_{2}}{\mathrm{sp}\sqrt{\frac{1}{n_{1}}+\frac{1}{n_{2}}}}$$

where ${\bar{x}}_{1}$ and ${\bar{x}}_{2}$ are the sample means, $n_{1}$ and $n_{2}$ are the sample sizes, and sp is the pooled standard deviation calculated from the two sample variances. The test statistic, *t*, was compared against a *t*‐distribution with $n_{1}+n_{2}-2$ degrees of freedom. A two‐tailed *p*‐value was calculated for each comparison, using a significance threshold of *α* = 0.05.

#### 2.6.2 Welch's two‐sample *t*‐test

Welch's t‐test was used to compare the means of two independent groups, particularly in cases where unequal variances or unequal sample sizes were expected. This test does not assume homogeneity of variance, making it more appropriate than the standard Student's *t*‐test under such conditions. The test statistic was calculated as:

(4) $$t=\frac{{\bar{x}}_{1}-{\bar{x}}_{2}}{\sqrt{\frac{s_{1}^{2}}{n_{1}}+\frac{s_{2}^{2}}{n_{2}}}}$$

with the degrees of freedom approximated using the Welch–Satterthwaite equation:

(5) $$v=\frac{{\left(\frac{s_{1}^{2}}{n_{1}}+\frac{s_{2}^{2}}{n_{2}}\right)}^{2}}{\frac{{\left(\frac{s_{1}^{2}}{n_{1}}\right)}^{2}}{n_{1}-1}+\frac{{\left(\frac{s_{2}^{2}}{n_{2}}\right)}^{2}}{n_{2}-1}}$$

A two‐tailed *p*‐value was used in all cases, with significance determined at the 0.05 level.

In these equations, ${\bar{x}}_{1}$ and ${\bar{x}}_{2}$ denote the sample means of groups 1 and 2, respectively, while $s_{1}^{2}$ and $s_{2}^{2}$ represent the corresponding sample variances. The terms $n_{1}$ and $n_{2}$ indicate the number of observations in each group. The numerator of the test statistic, ${\bar{x}}_{1}-{\bar{x}}_{2}$, represents the difference between the two‐sample means, and the dominator is the standard error of this difference, $\sqrt{\frac{s_{1}^{2}}{n_{1}}+\frac{s_{2}^{2}}{n_{2}}}$. The test statistic, t, was then evaluated against a t‐distribution with degrees of freedom, v, estimated using the Welch–Satterthwaite equation.

## 3 RESULTS AND DISCUSSION

### 3.1 Performance development of the Sartobind® Q operation

Due to the lack of commercially available micro‐scale depth filters, Sartobind® Q anion exchanger was selected as a micro‐scale mimic of the laboratory‐scale depth filtration operation to achieve material preparation for the subsequent polishing steps. Despite differences in the structure, selectivity, and binding mode, the comparable functionality for removal ensures only the negatively charged impurities are being targeted, specifically hcDNA and HCPs.^29^, ^30^ The removal of hcDNA is imperative to prevent IEX chromatographic fouling, ensuring high column performance and longer resin life, while increasing capacity and resolution. DNA may also compete for binding sites, and therefore, reduce the capacity for negatively charged impurities during the AEX operation, further highlighting the need to reduce the levels of hcDNA.^31^, ^32^ Consequently, Sartobind Q®, akin to the laboratory‐scale size‐ and charge‐based depth filter composed of cellulose fibers and DE, represents a method to achieve material preparation for the subsequent IEX operations. Tables 5 and 6 present a summary of the similarities and differences between Sartobind® Q and the size and charge‐based laboratory‐scale depth filter.

**TABLE 5:** Summary of the similarities between Sartobind® Q and the size‐ and charge‐based laboratory‐scale depth filter.

Aspect	Explanation	References
Target negatively charged impurities	Both are designed to remove negatively charged impurities, including hcDNA and HCPs	30
Mechanism	Despite differences in the structure and selectivity, both use electrostatic interactions for separation of negatively charged impurities. While the laboratory‐scale depth filter uses size exclusion to trap particulates, Sartobind® Q may also remove particles that are greater than the pore size (>3 μm)	17, 33
Single‐use friendly	Offered in disposable formats, making them ideal for single‐use bioprocessing	34, 35
Minimal footprint	Designed for high throughput with low hold‐up volumes. Suitable for continuous or intensified processing	34, 35

**TABLE 6:** Summary of the differences between Sartobind® Q and the size‐ and charged‐based laboratory‐scale depth filter.

Aspect	Sartobind® Q	Laboratory‐scale depth filter	References
Structure and flow path	Membrane sheets with convective flow	Porous fibrous media with tortuous flow path to enable depth capture	17, 33
Selectivity and binding mode	Highly selective for charged impurities, used in flowthrough or bind‐and‐elute modes	Less selective, capturing a wider range of process‐relate and product‐related impurities	13, 36
Micro‐scale availability	Available in a micro‐scale format which can be integrated within the automated platform	Not available in a micro‐scale format and cannot be integrated within the automated platform	34, 35
Typical application	Primarily used in polishing and viral clearance steps after primary capture	Typically used in clarification before ion exchange chromatography	36, 37

#### 3.1.1 Assessment of the dynamic binding capacity

Defining the DBC is an important aspect of chromatographic operations, which enables scale‐up, cost optimization, column sizing, process control, and most notably an increase in process efficiency.^38^ DBC refers to the maximum amount of a target molecule that a chromatographic resin can bind under specific flow conditions before breakthrough occurs.^39^, ^40^ Typically, the DBC is defined at the point where 10% of the target molecule appears in the column effluent, known as the 10% breakthrough.^40^

The experimental setup of the automated liquid handler differs from that of a traditional laboratory‐scale system in that solution transfer to the Sartobind® Q 96‐well plate is performed in a discrete, as opposed to a continuous manner. In this system, each of the eight channels of the LiHa aspirates liquid both within and between wells, which plays the role of an inlet and outlet pump.^11^, ^28^ As a result, liquid movement and separation of stationary and liquid phases is driven by centrifugation; therefore, binding occurs under non‐equilibrium, time‐limited conditions. Although flow rates are not precisely controlled and residence times may vary between wells, the system provides an approximation of dynamic binding capacity (DBC), or pseudo‐DBC, which can be used to guide process development and optimization.

**FIGURE 3:**
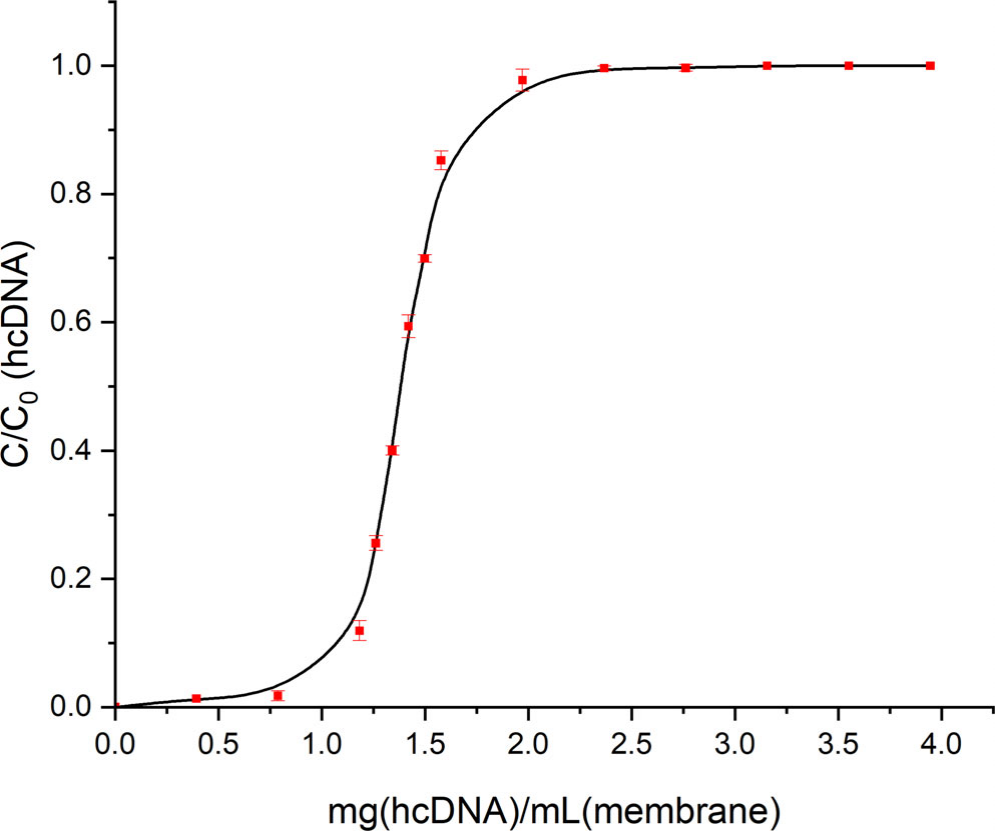
Breakthrough curve for hcDNA during column loading. The normalized concentration of hcDNA (*C*/*C*_0_) in the column effluent is plotted as a function of mg_hcdna_ mL_membrane_^−1^. The breakthrough point was defined as the effluent concentration (*C*) reaching 10% of the inlet concentration (*C*_0_). The sharp increase in *C*/*C*_0_ indicates the point at which the column becomes saturated with hcDNA, representing the breakthrough point. Error bars represent the standard deviation of triplicate analytical measurements.

The Sartobind® Q 96‐well plate was integrated into the micro‐scale platform, and the experimental workflow was designed accordingly. A total of 50 mL of the low pH VI process intermediate was processed through the Sartobind® Q membrane to determine hcDNA breakthrough. The resulting breakthrough curve is shown in Figure 3.

The pseudo‐DBC_10_ and pseudo‐DBC_50_ of Sartobind® Q for hcDNA are 1.11 mg_hcdna_ mL_membrane_^−1^ and 1.38 mg_hcdna_ mL_membrane_^−1^ and, respectively. These results indicate that the membrane exhibits a steep breakthrough profile, consistent with the breakthrough curve presented in Figure 3, and suggest efficient utilization of available binding sites until the point of saturation. These values fall within the range presented in literature. For example, Stone et al. reported a binding capacity before breakthrough of 0.8 mg_dna_ mL_resin_^−1^ at pH 6.0 and a conductivity of 15 mS/cm.^32^ In contrast, Sartorius Stedim Biotech GmbH (2015) reported a DBC of 5.6 mg_dna_ mL_resin_^−1^ at pH 7.25 and 25 mS/cm.^41^ Such variation in reported DBC values may be attributed to differences in sample composition, including DNA size distribution and the presence of competing HCPs, as well as experimental conditions, such as flow rate, pH, and conductivity. For example, Stone et al. demonstrated a three‐fold increase in the DNA binding capacity of a Mustang Q anion exchange membrane when operated at pH 8.0 and 2 mS/cm compared to pH 6.0 and 15 mS/cm.^32^

These findings not only validate the pseudo‐DBC values obtained in this study but also accentuate the suitability of Sartobind® Q for effective hcDNA removal, enabling tight control of hcDNA while providing a threshold for load volume during process development.

#### 3.1.2 Implementation of pathlength correction

Based on the defined pseudo‐DBC, the sample loading strategy was optimized to improve processing efficiency. As opposed to performing multiple loading steps within a single well of the 96‐well plate (each with a maximum capacity of 0.5 mL), the protocol was adjusted to utilize several wells to accommodate larger sample volumes. This allowed for processing of greater volumes in a single centrifugation step, thereby reducing overall operating time and improving throughput.

Additional modifications were made to the Sartobind® Q protocol after initial experiments yielded recoveries significantly below the acceptable range and those obtained in previous Sartobind® Q studies.^36^, ^42^, ^43^ The reduced recoveries were attributed to asynchronous dripping from individual wells of the 96‐well Sartobind® Q plate during centrifugation, which led to discrepancies between the volume dispensed onto the membrane and the volume collected. The offset also introduced variation in the pooled flowthrough material required for further downstream processing.

To correct for these volume differences, an additional task was added to the protocol. Following centrifugation, the 96‐well Sartobind® Q plate and its corresponding collection plate were placed on a BioShake module to recover any residual liquid from each well. Additionally, a pathlength correction (A900 vs. A977) was applied to the raw absorbance values.^44^ This correction calculated the pathlength and, therefore, volume in each collection well by assuming a K‐Factor of 0.173 prior to pooling. Table 7 shows a comparison between corrected and uncorrected recoveries of the pooled Sartobind® flowthrough. There is a significant difference (*p*‐value <0.05) between the recovery values, highlighting the importance of implementing a pathlength correction to the raw values following the Sartobind® Q operations.

**TABLE 7:** Comparison of calculated recoveries based upon corrected (pathlength correction) and uncorrected (no pathlength correction) A280 values following the Sartobind® Q operation.

Corrected	Uncorrected
92	86

### 3.2 Scale comparison of the micro‐scale Sartobind® Q and laboratory‐scale depth filtration operations

Following the processing of harvest material through protein A chromatography and low pH viral inactivation at both the laboratory and micro‐scale, the bioanalytical data for the process intermediates are presented in Table 8. This material represents the load material for both the laboratory‐scale depth filtration and micro‐scale Sartobind® Q operations.

**TABLE 8:** Analytical data characterizing the starting material used in the laboratory‐scale depth filtration and micro‐scale Sartobind® Q operations.

HMWC (%)		HCP (ppm)		hcDNA (ppm)	
Lab	Micro	Lab	Micro	Lab	Micro
7	7	2950	3100	300	420

This highlights the need for hcDNA clearance prior to processing through the IEX operations. This step is essential to prevent chromatographic fouling, ensuring high column performance.^31^, ^32^ Consequently, the performance of the micro‐scale Sartobind® Q will be evaluated based on its ability to remove the critical quality attribute (CQA), hcDNA, as well as HMWC and HCPs, which will be assessed against the performance of the laboratory‐scale depth filtration, and process specifications.

**FIGURE 4:**
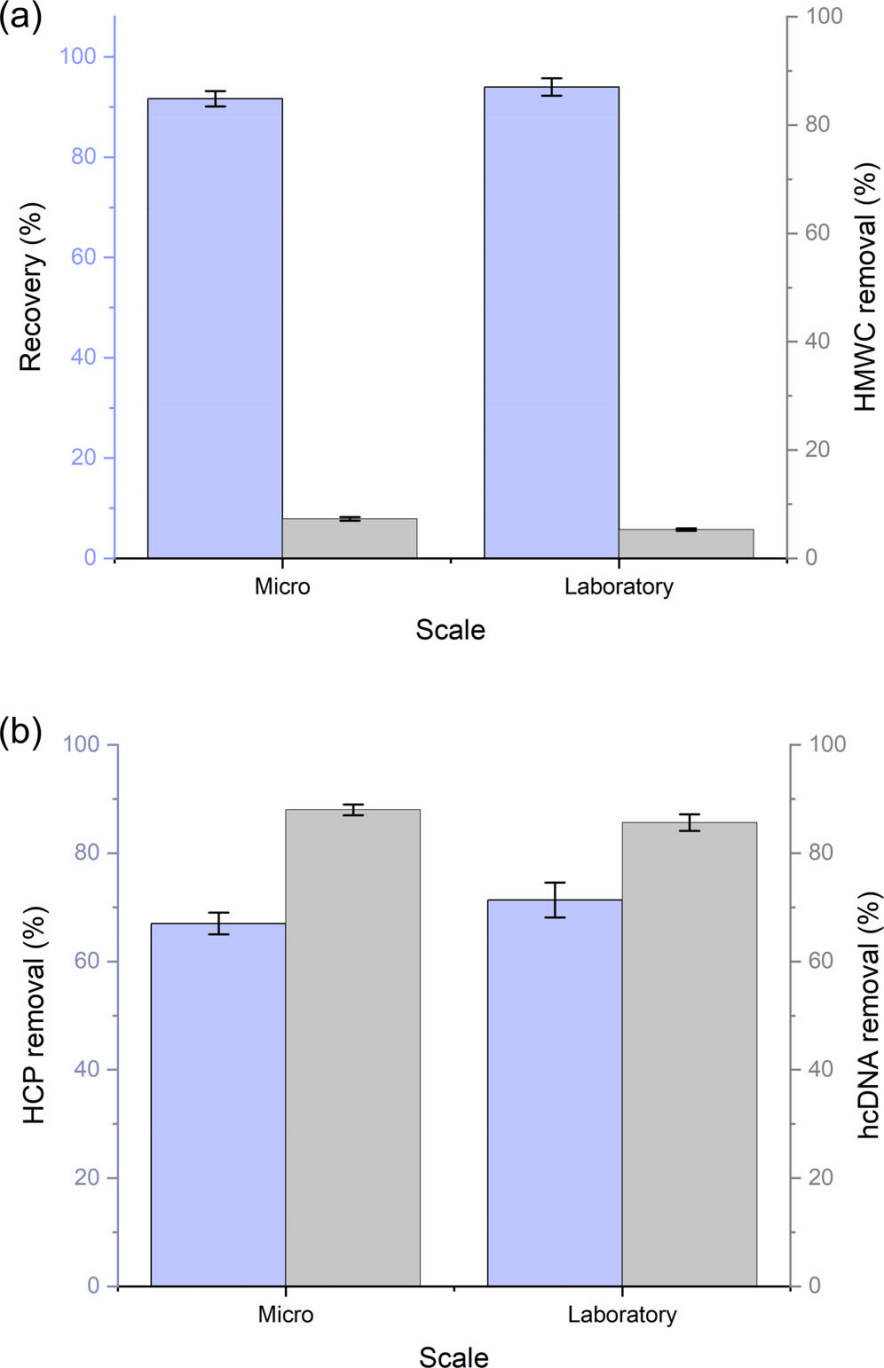
Comparison of the critical quality attributes (CQAs) between the micro‐scale Sartobind® Q and laboratory‐scale depth filter operations. (a) Recovery and high molecular weight content (HMWC) removal; (b) Host cell protein (HCP) removal and host cell DNA (hcDNA) before and after the depth filtration and Sartobind® Q operations for the laboratory‐scale and micro‐scale systems, respectively. Error bars represent the standard deviation from triplicate runs.

The starting material was placed onto the robotic liquid handler before executing the membrane plate workflow. The starting material was 0.5 mL of the low pH virus inactivated protein A eluate per well, and 8 wells were used to ensure sufficient material was processed for the downstream operations. The flowthrough was collected, pooled, and analyzed. The samples were measured at A280, A320, A900, and A977 before implementing a pathlength correction to the raw values. Figure 4a,b present the recovery, hcDNA, HCP, and HMWC of the micro‐scale Sartobind® Q and the laboratory‐scale depth filter.

As shown in Figure 4a both product recovery and HMWC removal at the micro‐scale were consistent with those observed at the laboratory scale, demonstrating strong comparability across scales. This operation exerted only a modest effect on HMWC clearance, with approximately 10% removal achieved in both cases, and according to Welch's *t*‐test (*p*‐value = 0.144), no statistically significant differences were observed for HHMWC removal between the two scales. In contrast, Figure 4b illustrates a more pronounced impact on HCP clearance, with removal efficiencies of around 70% at both scales, accompanied by the most substantial reduction in hcDNA levels within the purification sequence. The comparability of HCP and hcDNA clearance between the two scales was further confirmed by statistical analysis. A Welch's *t*‐test performed on HCP removal data indicated no significant difference between micro‐ and laboratory scale performance (*p*‐value = 0.13), while a Student's *t*‐test applied to hcDNA removal similarly found no significant difference between scales (*p*‐value = 0.09). Together, these results highlight the consistency of performance across scales and reinforce the critical role of this operation in HCP and hcDNA clearance prior to IEX.

The recovery results presented in Figure 4a are consistent with those reported in the literature. For example, Wang et al. employed Sartobind® Q within an integrated full‐membrane platform for the purification of three monoclonal antibodies and observed recoveries exceeding 95%.^42^ Similarly, Weaver et al. reported ~94% recovery when using Sartobind® Q in an antibody purification sequence.^36^ The minor deviation in the recoveries may be attributed to differences in pH and conductivity. Weaver et al. also reported a 2.5‐log removal of hcDNA and less than 0.1‐log removal of HCP under high salt and high pH conditions. While these findings highlight the primary role of Sartobind® Q in hcDNA clearance, the contribution to HCP removal is variable and dependent on the experimental conditions. For example, Nadal et al. reported 91% HCP removal at a lower pH and conductivity, suggesting that Sartobind® Q predominantly relies on Coulombic interactions for the removal of negatively charged contaminants.^36^, ^45^ Although the Sartobind® Q operations described here were implemented as part of a polishing strategy, the comparable recovery and impurity removal observed highlight the utility of micro‐scale Sartobind® Q for material preparation prior to the IEX operations.

### 3.3 Application of the micro‐scale platform to assess process intermediates

To gain insight into process interactions within the purification sequence, the integrated micro‐scale platform was employed to evaluate the material quality of process intermediates. The protein A eluate pool was incubated at various pH levels during the low pH VI operation to generate differing impurity profiles. These were subsequently processed through the remaining purification operations to assess the performance of the micro‐scale platform, particularly the ability of the Sartobind® Q step in impurity removal.

**FIGURE 5:**
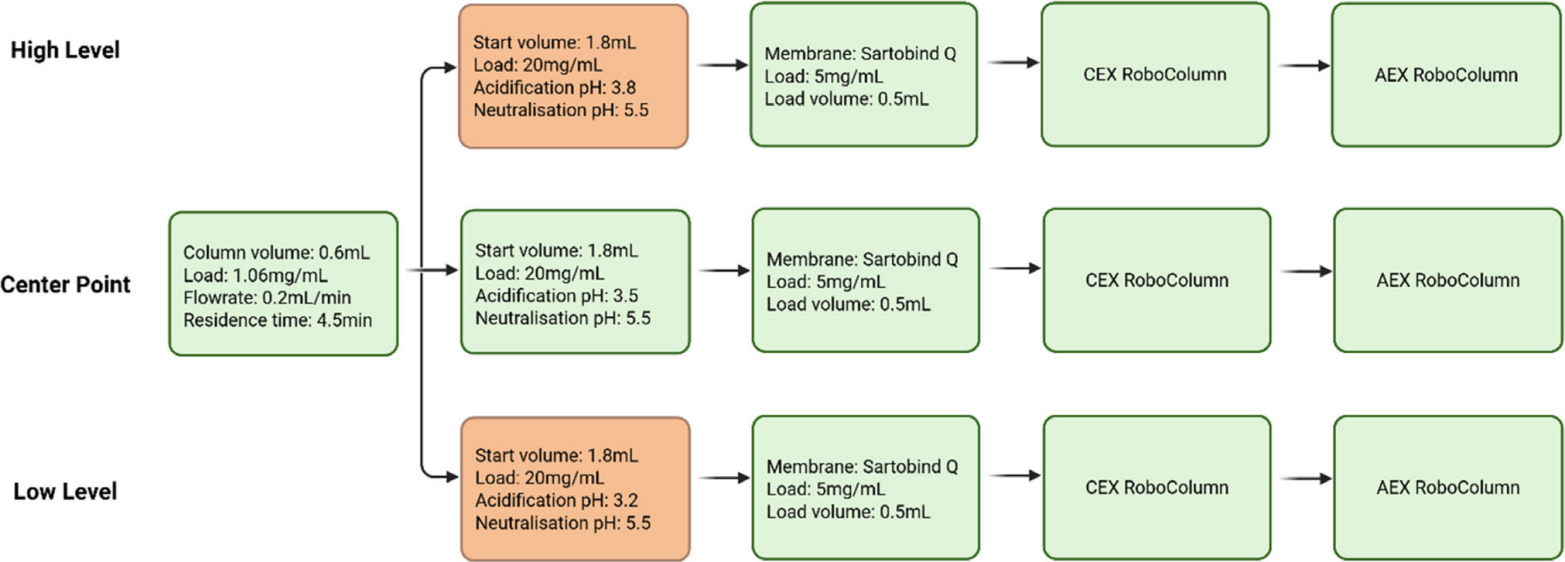
Process flowsheet of the monoclonal antibody purification sequence illustrating the application of the integrated micro‐scale platform to assess process interactions. The workflow was operated at three pH conditions: the centre point (pH 3.5), high level (pH 3.8), and low level (pH 3.2). These conditions were selected to generate varying impurity profiles in the process intermediate, which were subsequently processed through the downstream sequence to evaluate the impurity removal performance of the micro‐scale platform. Figure created with BioRender.com .

Following the protein A chromatography operation, the pooled protein A eluate was diluted 1:4 with protein A elution buffer prior to low pH VI. The volume of acidification solution added to the diluted eluate was varied to achieve target pH values of 3.2, 3.5, and 3.8. Each sample was incubated at the specified pH before being neutralized to pH 5.5. The resulting process intermediates were then processed in parallel through the downstream purification steps (Figure 5).

#### 3.3.1 Assessment of process intermediates to inform on process interactions

It is essential that the material output quality following each operation, and primarily after the entire process sequence, remains comparable to that achieved in the laboratory‐scale system and satisfies the predefined process specifications (Figure 1).

The starting harvest material (Table 9) was thawed and placed onto the robotic liquid handler prior to executing the protein A chromatography workflow. The purification process was carried out sequentially, with the material processed through each unit operation up to and including AEX. The eluted fractions from each step were collected and analyzed, with raw values adjusted using a pathlength correction. Analytical assays were conducted for each process intermediate, and the results for HMWC, HCP, hcDNA are summarized in Table 9 to highlight the impurity removal from each operation of the purification process. The laboratory‐scale system is also presented for comparison.

**TABLE 9:** Monoclonal antibody concentration and analytical data of harvest material which represents the starting material for the purification sequence.

Protein concentration (mg/mL)	HMWC (%)	HCP (ppm)	hcDNA (ppm)
1.3	18	100,000	1600

As presented in Table 10, the sequential purification process, from protein A chromatography through to AEX, resulted in effective and progressive reduction of both product‐ and process‐ related impurities, including HMWC, HCP and hcDNA. Additionally, comparative evaluation of laboratory‐scale and micro‐scale systems revealed high consistency in performance across all operations. While antibody recovery data are not shown, it is important to note that recoveries met predefined process specifications and were comparable between the two scales throughout the entire purification sequence.

**TABLE 10:** Summary of impurity clearance across the monoclonal antibody purification process at laboratory‐ and micro‐scale.

Protein A chromatography						
	HMWC (%)		HCP (ppm)		hcDNA (ppm)	
	Lab	Micro	Lab	Micro	Lab	Micro
	7	8	4500	4700	500	500

Low pH viral inactivation						
Hold pH	HMWC (%)		HCP (ppm)		hcDNA (ppm)	
	Lab	Micro	Lab	Micro	Lab	Micro
**3.2**	9	8	2700	2900	350	390
**3.5**	5	5	2900	3000	370	400
**3.8**	3	2	2600	2500	410	380

*Depth filtration* (Laboratory‐scale) and *Sartobind® Q* (Micro‐scale)						
Hold pH	HMWC (%)		HCP (ppm)		hcDNA (ppm)	
	Lab	Micro	Lab	Micro	Lab	Micro
**3.2**	8	8	*900*	*1100*	< *L.O.Q*	< *L.O.Q*
**3.5**	5	4	*900*	*1100*	< *L.O.Q*	< *L.O.Q*
**3.8**	2	2	*1000*	*1000*	< *L.O.Q*	*< L.O.Q*

Cation exchange chromatography						
Hold pH	HMWC (%)		HCP (ppm)		hcDNA (ppm)	
	Lab	Micro	Lab	Micro	Lab	Micro
**3.2**	2	2	50	70	< L.O.Q	< L.O.Q
**3.5**	1	1	50	60	< L.O.Q	< L.O.Q
**3.8**	1	1	30	40	< L.O.Q	< L.O.Q

Anion exchange chromatography						
Hold pH	HMWC (%)		HCP (ppm)		hcDNA (ppm)	
	Lab	Micro	Lab	Micro	Lab	Micro
**3.2**	0.2	0.2	< L.O.Q	< L.O.Q	< L.O.Q	< L.O.Q
**3.5**	0.6	0.8	< L.O.Q	< L.O.Q	< L.O.Q	< L.O.Q
**3.8**	0.6	0.8	< L.O.Q	< L.O.Q	< L.O.Q	< L.O.Q

*Note*: Critical quality attributes (CQAs) including high molecular weight species (HMWC, %), host cell protein (HCP, ppm), and host cell DNA (hcDNA, ppm) were measured following each operation: Protein A chromatography, low pH viral inactivation (VI) at varying hold pH values (3.2, 3.5, and 3.8), depth filtration or Sartobind® Q, cation exchange chromatography (CEX), and anion exchange chromatography (AEX). L.O.Q refers to the limit of quantification of the analytical assay.

Low pH VI serves as the dedicated virus reduction step in the purification process and is typically achieved by holding the process intermediate at pH 3.5 for a defined duration.^46^ However, this step is commonly associated with an increased risk of protein aggregation, requiring careful selection of the hold pH to balance viral clearance with product stability.^47^, ^48^ The antibody used in this study is aggregation‐prone; therefore, a 1:4 dilution was performed prior to viral inactivation to reduce conductivity and minimize aggregation. The CQA associated with this step is the level HMWC. To investigate the impact of pH on aggregation and impurity levels, the operation was conducted at pH values of 3.2, 3.5, and 3.8. A trend of reduced HMWC with increasing pH was observed, decreasing from 9% to 3% in the laboratory‐scale system and from 8% to 2% in the micro‐scale platform. These findings suggest greater aggregation at lower pH values, consistent with previous studies involving this molecule.^11^ HCP and hcDNA levels showed minor fluctuations across the pH conditions. The variations across scales may be attributed to differences in mixing dynamics during the incubation phase, as the micro‐scale system's mechanism may not fully replicate that of the laboratory‐scale setup.

**FIGURE 6:**
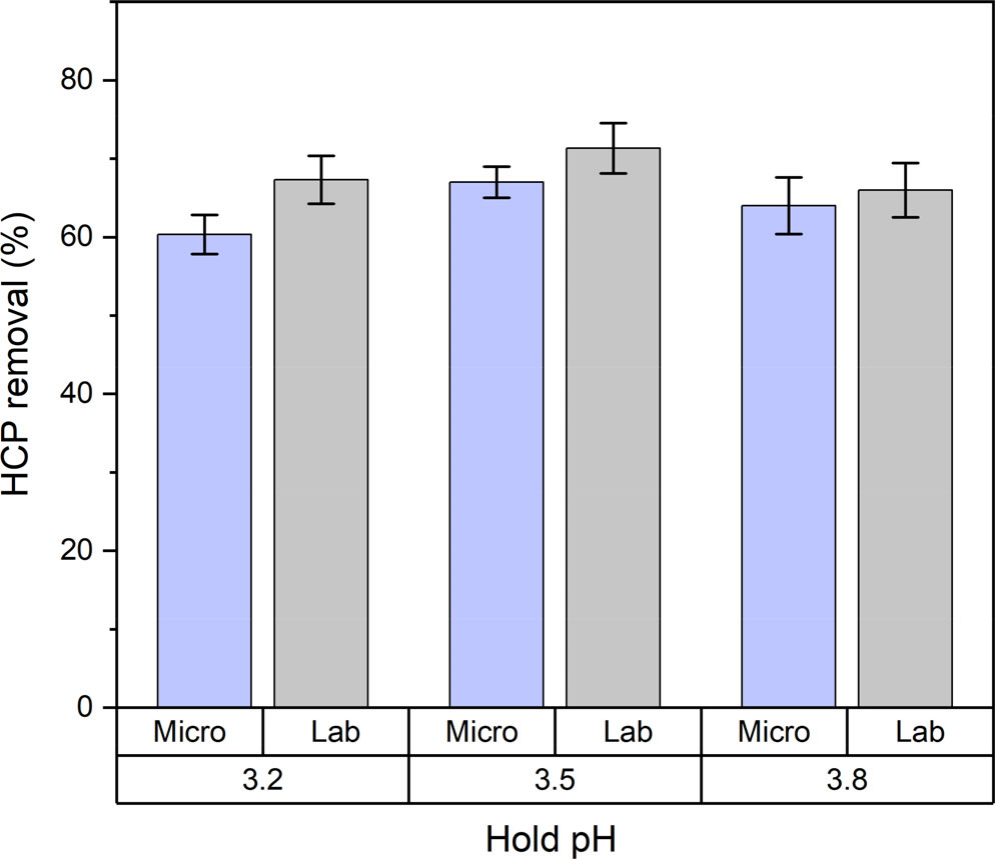
Host cell protein (HCP) removal following the depth filtration step in the laboratory‐scale system and Sartobind® Q in the micro‐scale platform, across varying hold pH conditions during the low pH VI operation. Error bars represent the standard deviation from triplicate runs.

**FIGURE 7:**
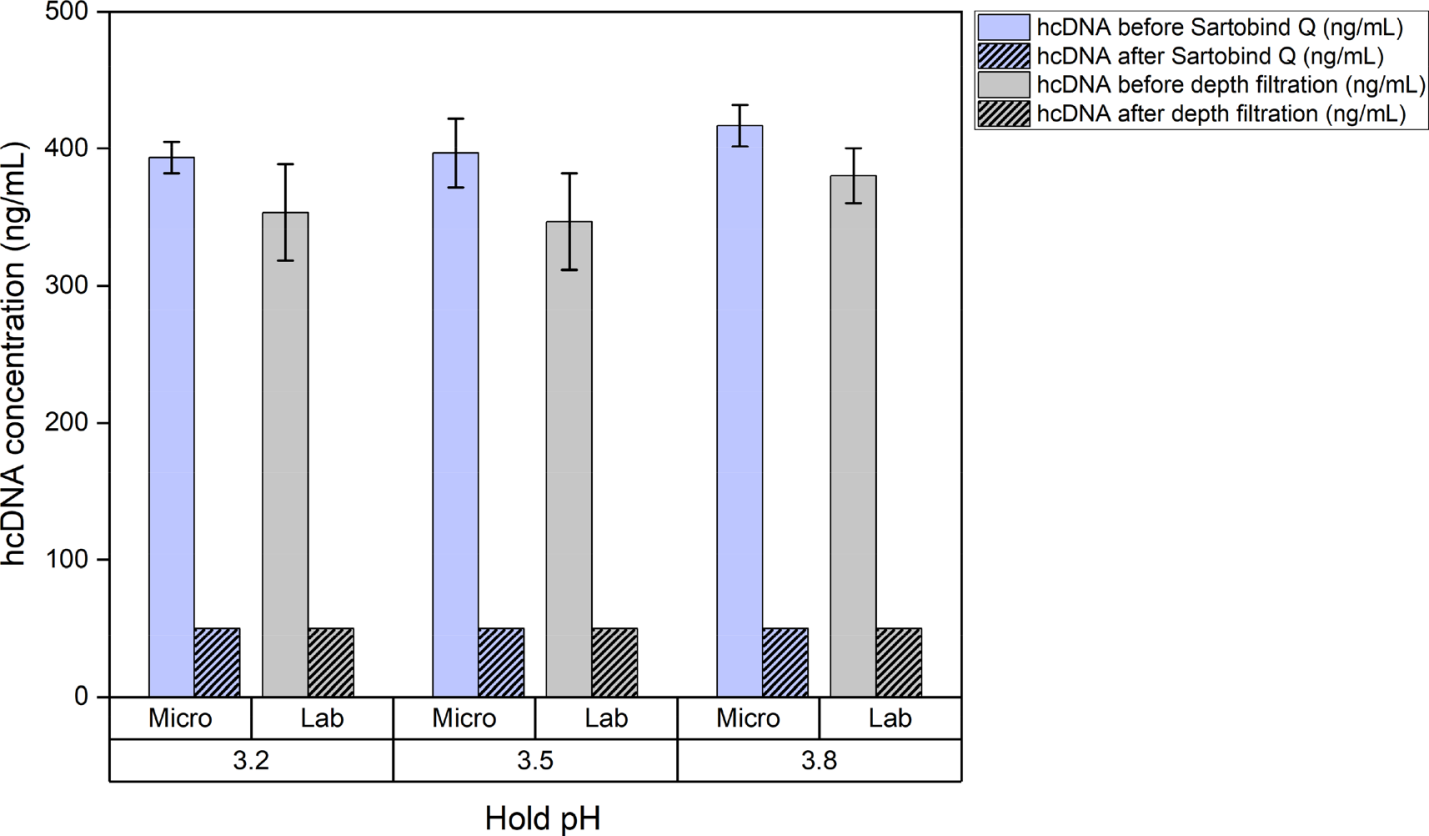
Host cell DNA (hcDNA) concentrations before and after the depth filtration step in the laboratory‐scale system and Sartobind® Q in the micro‐scale platform, following low pH viral Inactivation (VI) at pH 3.2, 3.5, and 3.8. The hcDNA concentration was reduced to below the limit of quantification (L.O.Q) across all tested conditions. Error bars represent the standard deviation from triplicate runs.

Depth filtration, or Sartobind® Q in the micro‐scale platform, serves as an intermediate purification step for the removal of residual process‐related contaminants, with hcDNA and HCP identified as the key CQAs. This operation resulted in a consistent reduction of HCP, with approximately 65% removal observed in the laboratory‐scale system and 60% in the micro‐scale setup across all tested conditions (Figure 6). Importantly, complete clearance of hcDNA to below the L.O.Q. was achieved in both systems (Figure 7), demonstrating the effectiveness of this step in DNA removal. Although HMWC levels showed only marginal reductions at this stage, all CQAs remained within predefined process specifications. The comparability between laboratory‐ and micro‐scale performance further highlights the suitability of Sartobind® Q for this stage of the purification process.

CEX is employed for the removal of both product‐ and process‐related impurities, with HMWC and HCP identified as the key CQAs for this operation. This step delivered substantial purification, particularly with respect to HMWC and HCP clearance. A 75% reduction in HMWC levels was achieved at pH 3.2 across both the laboratory‐ and micro‐scale platforms, underscoring the capability of CEX to effectively reduce aggregates, even in the context of elevated starting levels. HCP clearance exceeded 90% under all tested conditions for both scales, further demonstrating the robustness of this step. Collectively, these results confirm the efficiency of CEX in impurity polishing and aggregate removal, with all CQAs maintained within predefined process specifications.

AEX constitutes the final operation in the purification sequence and is employed for the removal of residual product‐ and process‐related impurities, with HMWC and HCP identified as the key CQAs. In both the laboratory‐ and micro‐scale systems, HCP was reduced to below the limit of detection, while HMWC levels were further decreased by 90% at pH 3.2. These results underscore the effectiveness of the final polishing step in removing aggregates, even in the context of elevated starting levels. All final product CQAs were within predefined process specifications and were comparable to impurity clearance levels reported in previously established mAb purification processes.^42^

The progressive reduction in impurities observed across each unit operation highlights the overall effectiveness of the process for mAb purification. Importantly, the close alignment in impurity clearance between the laboratory‐ and micro‐scale systems, across all operations and varying impurity profiles, demonstrates the robustness and reliability of the scaled‐down approach for process development. The successful integration of the Sartobind® Q operation within the micro‐scale platform further highlights its suitability as a preparatory step for subsequent IEX steps, as is depth filtration in the laboratory‐scale process. Collectively, these findings support the utility of the integrated, automated micro‐scale platform not only for accelerating process development and enhancing process understanding but also as a rapid assessment tool to optimize mAb purification sequences.

### 3.4 Evaluation of the integrated micro‐scale platform

To evaluate the utility of the integrated micro‐scale platform, this study focused on its application in assessing process intermediates, with particular emphasis on total run time for each unit operation and the complete purification sequence. This enables a direct comparison between the micro‐scale platform and a conventional laboratory‐scale system in terms of process efficiency and throughput.

A process capability study was conducted to explore the impact of varying hold pH conditions, 3.2, 3.5, and 3.8, during the low pH VI step, obtaining distinct impurity profiles, as described in the previous sections. The outcomes of the full purification sequence under these conditions were subsequently assessed to determine the process edge of failure. The micro‐scale platform was evaluated as a fully integrated system to accurately reflect its operational utility, thereby providing a realistic assessment of its potential for high‐throughput process development.

**FIGURE 8:**
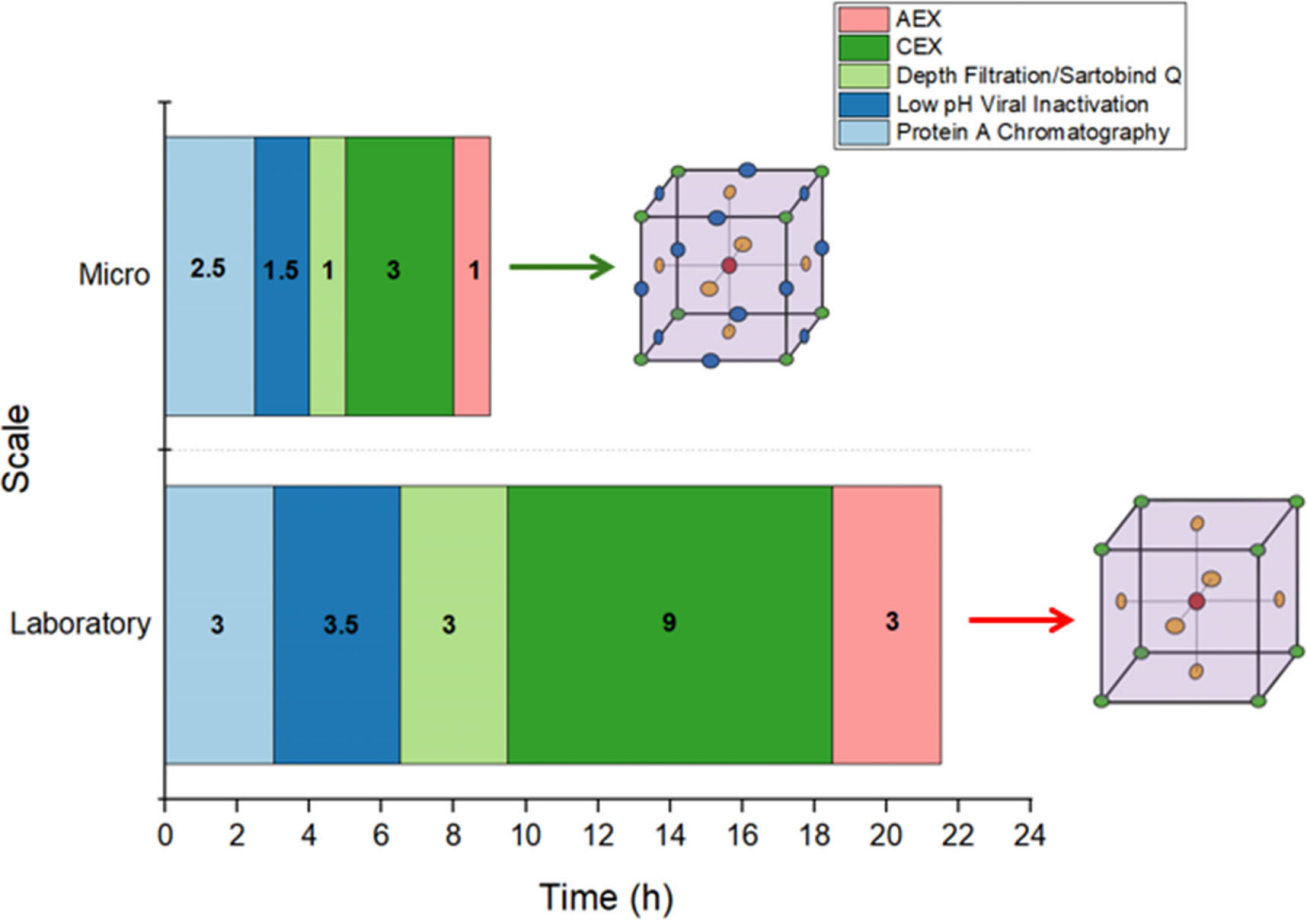
Stacked bar chart illustrating the timepoints and durations of each unit operation for the laboratory‐scale and micro‐scale systems. The duration of each operation is indicated within the bars. The accompanying design matrices exemplify the scope and feasibility of design of experiment approaches applicable to each system, based on their respective process durations.

Figure 8 illustrates the sequence of unit operations and corresponding time points in both laboratory‐scale and micro‐scale purification workflows, enabling a comparative evaluation of process efficiency. The laboratory‐scale process spans 21.5 h, whereas the micro‐scale process takes only 9 h—a 58% reduction in total process time. This improvement is primarily due to the parallelization capabilities of the micro‐scale platform.^49^ For example, the Sartobind® Q step demonstrates a three‐fold time reduction compared to the depth filtration operation, with consistent gains observed in the micro‐scale CEX and AEX operations. The low pH VI step also benefits from this approach, showing a 57% reduction in operation time at the micro‐scale, relative to the laboratory‐scale. Overall, this parallelization enables faster progression through the purification sequence by allowing simultaneous processing of the three different process intermediates from the hold pH conditions, rather than sequentially, thereby enabling tighter scheduling of unit operations.

An additional advantage of the micro‐scale platform is its ability to generate increased analytical data within a shorter timeframe, without compromising data quality or integrity. For example, 9 chromatograms can be obtained in each of the micro‐scale CEX and AEX operations, compared to just 3 chromatograms at the laboratory scale. Furthermore, analytical data for process intermediates following Protein A chromatography, low pH VI, and Sartobind® Q is acquired faster than in the corresponding laboratory scale operations. This enables more frequent monitoring and faster decision‐making, supporting a more agile and responsive development workflow.

The micro‐scale platform outperforms the laboratory‐scale process in terms of time efficiency, data acquisition frequency, and process insight. This translates into a more compact, efficient workflow with increased analytical throughput in less than 50% of the time. These productivity gains can be leveraged to explore a broader design space within the same timeframe required by laboratory‐scale experiments. For example, the number of experimental levels assessed in a DoE study can be increased, as illustrated by the design matrices in Figure 8. Micro‐scale experiments demand fewer resources and less time, allowing for greater parallelization and significantly higher experimental throughput, ultimately leading to greater process understanding for process development and optimization.

## 4 CONCLUSIONS AND FUTURE OUTLOOK

This study demonstrates the successful integration of a Sartobind® Q anion exchange adsorber, serving as a depth filtration mimic, within an automated micro‐scale platform for monoclonal antibody purification. The micro‐scale workflow, designed using Robotools and implemented on the Tecan Freedom Evo 200, achieved performance equivalent to laboratory‐scale operations in terms of recovery, hcDNA clearance, HCP removal, and HMWC reduction for the Sartobind® Q and depth filtration operations, respectively. The ability to achieve consistent impurity clearance across varying process intermediate profiles, which was obtained by holding the process intermediate at different pH values during the low pH VI operations, highlights the success of the integrated micro‐scale platform, which can be utilized to inform on process development and optimization.

The utility of the integrated micro‐scale platform was assessed and compared with a typical laboratory‐scale system by evaluating the time required to conduct the study presented in this research. The advantages of the integrated platform were realized through the miniaturization and parallelization of experimentation. This results in lower time demands, increased labor efficiency, and greater productivity compared to the laboratory‐scale system. For example, the entire experiment can be conducted in 9 hours, as opposed to 21.5 h for the micro‐scale and laboratory‐scale systems, respectively. This highlights the significant time and labor savings that can be achieved when leveraging the integrated platform beyond chromatographic operations.

The integrated micro‐scale platform provides a foundation for more holistic mAb purification strategies. Future expansion to include additional non‐chromatographic operations, such as on‐deck pH monitoring during low pH viral inactivation, in combination with Design of Experiments (DoE) and process analytical technology (PAT), will further enhance its capability as a comprehensive high‐throughput process development system.

## AUTHOR CONTRIBUTIONS

Conceptualization: Paras Sharma, Lars Robbel, Michael Schmitt, Daniel G. Bracewell. Experimental work: Paras Sharma. Analytical work: Petra Sebastian. Writing – original draft preparation: Paras Sharma. Supervision: Lars Robbel, Michael Schmitt, Daniel G. Bracewell. Funding acquisition: Lars Robbel, Michael Schmitt, Daniel G. Bracewell.

## FUNDING INFORMATION

This work was supported by EPSRC Doctoral Training Partnership (DTP) (EP/T517793/1) and CSL Innovation GmbH.

## CONFLICT OF INTEREST STATEMENT

The authors do not declare any conflicts of interest.

## ATA AVAILABILITY STATEMENT

The data that supports the findings of this study is available from the corresponding author upon reasonable request.

## Data Availability

The data that support the findings of this study are available from the corresponding author upon reasonable request.
